# Treatment of advanced gastrointestinal tumors with genetically modified autologous mesenchymal stromal cells (TREAT-ME1): study protocol of a phase I/II clinical trial

**DOI:** 10.1186/s12885-015-1241-x

**Published:** 2015-04-08

**Authors:** Hanno Niess, Jobst C von Einem, Michael N Thomas, Marlies Michl, Martin K Angele, Ralf Huss, Christine Günther, Peter J Nelson, Christiane J Bruns, Volker Heinemann

**Affiliations:** 1 Department of General, Visceral, Transplantation, Vascular and Thoracic Surgery, Hospital of the University of Munich, Munich, Germany; 2 Department of Medical Oncology and Comprehensive Cancer Center, Hospital of the University of Munich, Munich, Germany; 3 Apceth GmbH and Co. KG, Munich, Germany; 4 Medizinische Klinik und Poliklinik IV, Campus Innenstadt, Klinikum der Universitaet Muenchen, Arbeitsgruppe Klinische Biochemie, Munich, Germany; 5 Department of Surgery, Hospital of the University of Magdeburg, Magdeburg, Germany

**Keywords:** Genetically engineered mesenchymal stromal cells, MSC, Gene therapy, Cell therapy, Suicide gene therapy, Clinical trial, HSV-Tk, Ganciclovir

## Abstract

**Background:**

Adenocarcinoma originating from the digestive system is a major contributor to cancer-related deaths worldwide. Tumor recurrence, advanced local growth and metastasis are key factors that frequently prevent these tumors from curative surgical treatment. Preclinical research has demonstrated that the dependency of these tumors on supporting mesenchymal stroma results in susceptibility to cell-based therapies targeting this stroma.

**Methods/Design:**

TREAT-ME1 is a prospective, uncontrolled, single-arm phase I/II study assessing the safety and efficacy of genetically modified autologous mesenchymal stromal cells (MSC) as delivery vehicles for a cell-based gene therapy for advanced, recurrent or metastatic gastrointestinal or hepatopancreatobiliary adenocarcinoma. Autologous bone marrow will be drawn from each eligible patient after consent for bone marrow donation has been obtained (under a separate EC-approved protocol). In the following ~10 weeks the investigational medicinal product (IMP) is developed for each patient. To this end, the patient’s MSCs are stably transfected with a gamma-retroviral, replication-incompetent and self-inactivating (SIN) vector system containing a therapeutic promoter - gene construct that allows for tumor-specific expression of the therapeutic gene. After release of the IMP the patients are enrolled after given informed consent for participation in the TREAT-ME 1 trial. In the phase I part of the study, the safety of the IMP is tested in six patients by three treatment cycles consisting of re-transfusion of MSCs at different concentrations followed by administration of the prodrug Ganciclovir. In the phase II part of the study, sixteen patients will be enrolled receiving IMP treatment. A subgroup of patients that qualifies for surgery will be treated preoperatively with the IMP to verify homing of the MSCs to tumors as to be confirmed in the surgical specimen.

**Discussion:**

The TREAT-ME1 clinical study involves a highly innovative therapeutic strategy combining cell and gene therapy and is conducted at a high level of pharmaceutical quality ensuring patient safety. This patient-tailored approach represents the first clinical study worldwide utilizing genetically engineered MSCs in humans.

**Trial registration:**

EU Clinical Trials Register/European Union Drug Regulating Authorities Clinical Trials Database number: 2012-003741-15

## Background

Cancers arising from the digestive system are high in incidence and account for a major proportion of cancer-related deaths [1]. Adenocarcinomas originating here are suspected to have a common embryonic origin, which lies in the endo-mesodermal germ layer. Thus, these tumors also share morphologic characteristics. Clinical problems arising during the treatment course of patients affected by these cancers include high rates of tumor recurrence, metastasis, resistance to chemotherapeutic agents/radiation therapy and locally advanced tumor growth often leading to the impossibility of performing potentially curative surgical resection. Among the most common themes seen in adenocarcinomas of different origin within the digestive system is the observation that all comprise a supporting tumor stroma that accompanies the malignant epithelial tumor cells and that is crucial for advancing tumor growth beyond a microscopic state [2]. The cellular composition of the stroma mostly involves a mononuclear cell infiltrate, fibroblasts, blood and lymphatic endothelial cells, pericytes and other cell types that mainly sustain the supply of tumor cells with oxygen and nutrients but also stimulate tumor cell proliferation in a paracrine fashion [3]. Recent strategies focusing cancer therapy on the stromal compartment of tumors by different therapeutic means has yielded high efficacy in suppressing overall tumor growth [4].

It is a well-recognized concept that growing malignant tumors resemble a chronic wound with regard to the microenvironment. Mesenchymal stromal cells (MSC) generally have the capability to undergo “homing” to sites of inflammation within the human body where they carry out their physiological functions. The term “homing” describes a complex, multi-step process similar to that seen in leukocyte trafficking. Upon stimulation, MSCs are released from their tissue of origin - usually the bone marrow -, enter the circulation, extravasate, migrate towards spots of inflammation and then undergo differentiation according to the respective stimuli present [5]. Through their differentiation potential, cell-to-cell signaling capabilities and ability to produce soluble factors, MSCs are involved in tissue repair and regeneration. However - presumably by the same mechanisms seen in inflammation-related homing - MSCs are susceptible to becoming encroached by tumors, more specifically so by contributing to the tumor stroma.

Making use of the tumor tropism observed in MSCs has been the general concept behind numerous preclinical therapeutic applications. To induce anti-tumor effects, MSCs have been transduced with interferon-γ, interferon-β, TNF-related apoptosis-inducing ligand (TRAIL), or CX3CL1 expressing vectors, respectively [6-8]. These applications have resulted in effective inhibition of tumor growth in animal studies. Our group has previously produced and successfully applied MSCs that have been stably transfected with vectors containing a gene construct ensuring tumor-specific expression of thymidine kinase of the herpes simplex virus (*HSV-Tk*) by MSCs in different preclinical animal studies [9-11]. Linking its expression to the CCL5/RANTES promoter or the Tie2 promoter/enhancer ensured tumor-specific expression of the suicide gene. With these constructs, transgene expression was achieved in the context of angiogenesis or the fibroblast compartment of tumor stroma, respectively. In the therapeutic construct, the thymidine kinase present in transfected cells phosphorylates the prodrug ganciclovir (GCV) to ganciclovir triphosphate, which acts as a competitive inhibitor of deoxyguanosine triphosphate, causes inhibition of DNA polymerases and subsequently drives affected cells into apoptosis. As a bystander effect, the toxic ganciclovir triphosphate may also reach surrounding cells through gap junctions and phagocytosis [12] and may thereby cause apoptosis.

In previous studies, treatment was attempted by direct inoculation of the viral transgene *HSV-TK* into cancer cells followed by GCV administration [13,14]. However, most attempts of direct transfection of tumor cells *in vivo* have had to deal with the problem of insufficient gene delivery to the target cells. In our preclinical animal studies, where we combined gene and MSC therapy, we were able to confirm tumor stroma as the main target of this form of therapy. Profound effects on primary tumor growth and formation of metastasis were observed [9-11].

**Figure 1:**
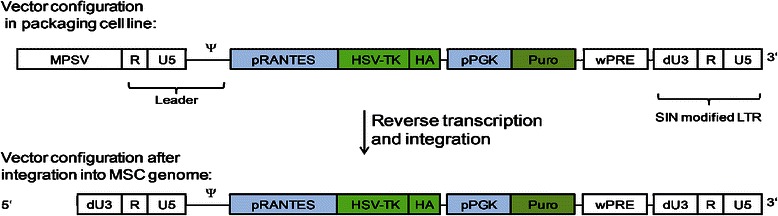
**Configuration of the retroviral vector pEMTAR-bi.RANTES.tk used to transfect primary MSCs to generate the IMP MSC_apceth_101.** MPSV: myeloproliferative sarcoma virus-promoter γ-retroviral R-region, U5: γ-retroviral U5-region, Ψ: Packaging signal, pRANTES: human RANTES promoter, HSV-TK: Herpes simples Thymidine kinase, HA: Hemagglutinin epitope, pPGK: human Phosphoglycerate Kinase promoter, Puro: Puromycin resistance gene, wPRE: woodchuck Hepatitis post-transcriptional regulatory element, dU3: deleted γ-retroviral U3-region, SIN: self-inactivating.

The IMP (designated MSC_apceth_101) used in this planned clinical phase I/II study (TREAT-ME1 trial) consists of human genetically-engineered autologous MSC. Scientifically, these cells classify as MSCs as specified in the position paper by Dominici and coworkers for the International Society for Cellular Therapy [15]. They exhibit the typical properties that identify MSCs: they form a typical fibroblast-like morphology and express surface markers CD73, CD90 and CD105 while lacking expression of CD45 and CD34. The local government and the national regulatory authority “Paul-Ehrlich-Institute” have issued a GMP-Manufacturer’s license for the IMP MSC_apceth_101. The process can be summarized as follows: Bone marrow is collected from each patient from the iliac crest and seeded in culture vessels. Plastic adherent MSC are expanded in a proprietary, xenofree medium while non-adherent hematopoietic cells are lost over time due to media exchanges (This described cultivation process is currently also used to generate native, unmodified form MSC (“MSC-Apceth”) for another clinical trial (EudraCT-No.: 2010- 021821–10)). At the time of the first passage, MSC are genetically modified by transduction using a gamma-retroviral SIN-vector (Figure 1). Transduced cells are cultured for several days and then subjected to puromycin selection to enrich transduced cells in culture. The transduced and selected cells are expanded until the clinical necessary dose has been generated. MSC_apceth_101 maintain their MSC properties and are further characterized according to GMP standards. Preceding the initiation of the TREAT-ME1 trial, pilot studies were performed after approval of the local ethics committee in which feasibility of MSC retrieval, transfection and expansion to a sufficient cell count in patients that underwent surgery for advanced tumors was shown.

## Methods/study design

### Study rationale

The highly innovative therapy tested in the TREAT-ME1 study utilizes the patient’s own MSCs to combine cell and gene therapy at a high pharmaceutical quality level. The specific mode of action of the suicide gene engineered into MSC_apceth_101 has the purpose of limiting the toxic gene therapy to the tumor and thus this therapy may display a favorable tolerability and safety profile. Presumably, this yields a high impact for all patients suffering from advanced tumors as the side effects of the standard and second-/third line therapies can significantly hamper completion of full treatment with these drugs. This in turn may provoke development of chemoresistance in tumors. The main rationale for the actively recruiting TREAT-ME1 trial is to demonstrate good tolerability of treatment with MSC_apceth_101 in combination with GCV and to assess a potential therapeutic benefit based on RECIST criteria [16].

### Manufacturing of IMP

The IMP is manufactured according to GMP requirements by apceth GmbH & Co KG (Munich, Germany). Bone marrow collected from each patient is seeded in cell factories (5–10 × 10^4^ white blood cells per cm^2^). Cells are cultured in a proprietary medium by apceth and passaged using Trypsin. At the first passage, cells are collected and retrovirally transduced. For this, MSCs are incubated with retroviral vector supernatant (Figure 1) at a multiplicity of infection (MOI) of 1 – 4 for 1 – 4 h to achieve a transduction efficacy of app. 2 – 30%. Transduced MSCs are cultured for 3 – 5 days until puromycin selection (3–5 days) is started to enrich for transduced cells. Transduced and enriched MSCs are then expanded until the clinical dose is reached. Cells are then detached, washed with PBS and re-suspended in cryo-protectant medium containing human serum albumin, DMSO and hydroxyethyl starch at a density of 5 × 10^6^ per ml. The cells are then cryopreserved in liquid nitrogen until they will be applied to the patient.

The following criteria/tests must *all* be fulfilled before the IMP can be released for the study:

- Absence of mycoplasma, aerobic and anaerobic bacteria and fungi,
- Expression of MSC markers of more than 90% of cells [15],
- Contamination with hematopoietic cells must be less than 10%,
- Viability of cells as tested by 7-AAD exclusion must be >80%,
- >75% of MSC must be positive for the transgene as tested by flow cytometry,
- Vector copy number must be between 0.7 and 3.8,
- GCV sensitivity must show that the IC_50_ of IMP with regard to GCV must be 2log below non-modified MSCs, which we consider a positive test for transgene functionality,
- Cells must show *no* anchorage independent growth in the according assay to rule out tumorigenic potential.

The retroviral vector supernatant was produced according to GMP requirements. A stable packaging cell line based on PG13 cells was generated as described elsewhere [17]. Supernatant was produced as described in [18]. The cell lines and all batches of vectors are tested for the absence of replication competent retroviruses (RCR).

### General aspects/ethics

TREAT-ME1 was designed in collaboration between the Department of General, Visceral, Transplantation, Vascular and Thoracic Surgery as well as the Department of Clinical Oncology of the University of Munich and the apceth GmbH &Co. KG based in Munich, Germany. It is an uncontrolled, single arm trial consisting of a phase I- followed by a phase II study. The local ethics committee of the University of Munich has approved the study and it has been registered at European Union Drug Regulating Authorities Clinical Trials Database/EU Clinical Trials Register (EudraCT/EU-CTR) with the registration number: 2012-003741-15 (https://www.clinicaltrialsregister.eu/ctr-search/trial/2012-003741-15/DE). All patients enrolled share a documented diagnosis of advanced, recurrent, or metastatic adenocarcinoma of the digestive system including esophageal, gastric, pancreatic, intrahepatic cholangiocarcinoma or colorectal carcinoma. To our knowledge, it is the first clinical study utilizing genetically modified MSCs in humans.

The bone marrow aspiration protocol with the title “Evaluation of the use of the patient's own bone marrow stromal cells (MSC) to develop and produce investigational medicinal products (IMP) based on autologous stromal cells for the treatment of solid tumors” is separate from the clinical trial protocol of the TREAT-ME1 study. All patients are informed about the sequence of the study and written consent to the bone marrow donation all procedural risks as well as the possibility of a too low cell yield to participate in the study, is obtained from all patients prior to the procedure. The bone marrow donation takes place in the operating theatre of the Department of Surgery, University of Munich.

The bone marrow for the production of MSC_apceth_101 is aspirated for all patients at least 10 weeks (10 weeks is the approximate production time for MSC_acpeth_101) before the intended administration of the IMP. The patient is screened for bone marrow donation and additionally only informed about the participation in the clinical trial and when the IMP is manufactured and released for use. Once the patient specific IMP is released, the patient will be invited by the investigator to the study site and will be officially screened for study participation. Without IMP availability, the patient will be not screened. If the IMP cannot be used for the clinical trial the patient will be asked, by signing a document, if the bone marrow can be used for scientific purposes. The responsible ethics committee has approved the protocol for bone marrow donation along with the overall study workflow.

This study complies with the Declaration of Helsinki in its most recent German version, the Medical Association's professional code of conduct, the principles of Good Clinical Practice (GCP) guidelines and the Federal Data Protection Act. The trial will also be carried out in keeping with local legal and regulatory requirements. Informed consent, during which a physician will explain the nature, scope, and possible consequences of the trial to the patient, is obtained from each patient in oral and written form before enrollment in the study. Apart from the bone marrow donation, which is performed under a separate protocol, the investigator will not assume any actions specifically required only for this clinical trial until valid consent has been obtained.

### Study design

**Figure 2:**
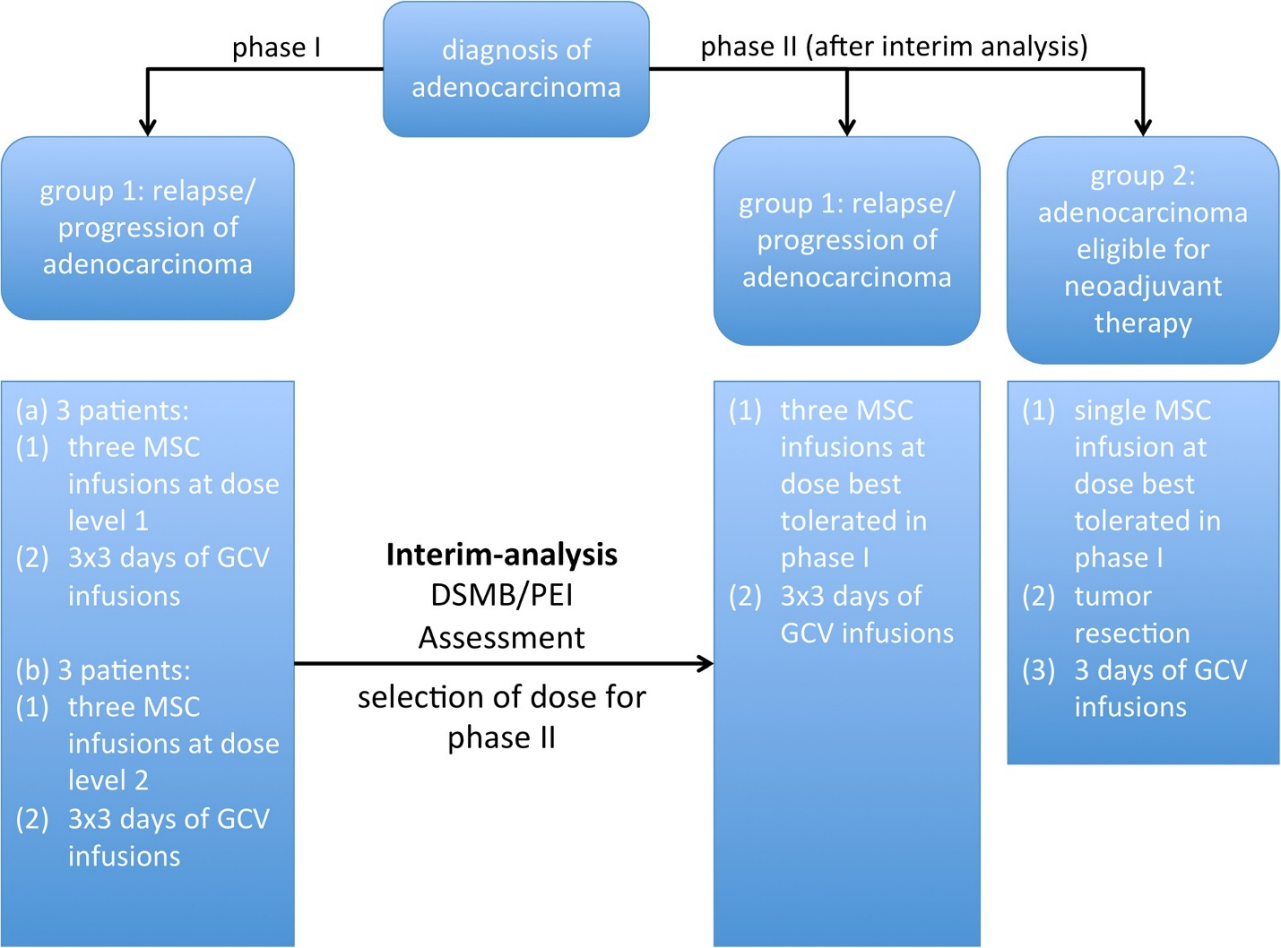
**Stages of the TREAT-ME1 study.** The TREAT-ME1 trial will be conducted in two stages, which are interrupted by an interim-analysis. Phase II will include two patient groups. Group 1 will be treated by the same treatment schedule as in phase I. Group 2 will consist of patient that are eligible for surgical resection of the tumor but that need neoadjuvant therapy.

**Figure 3:**
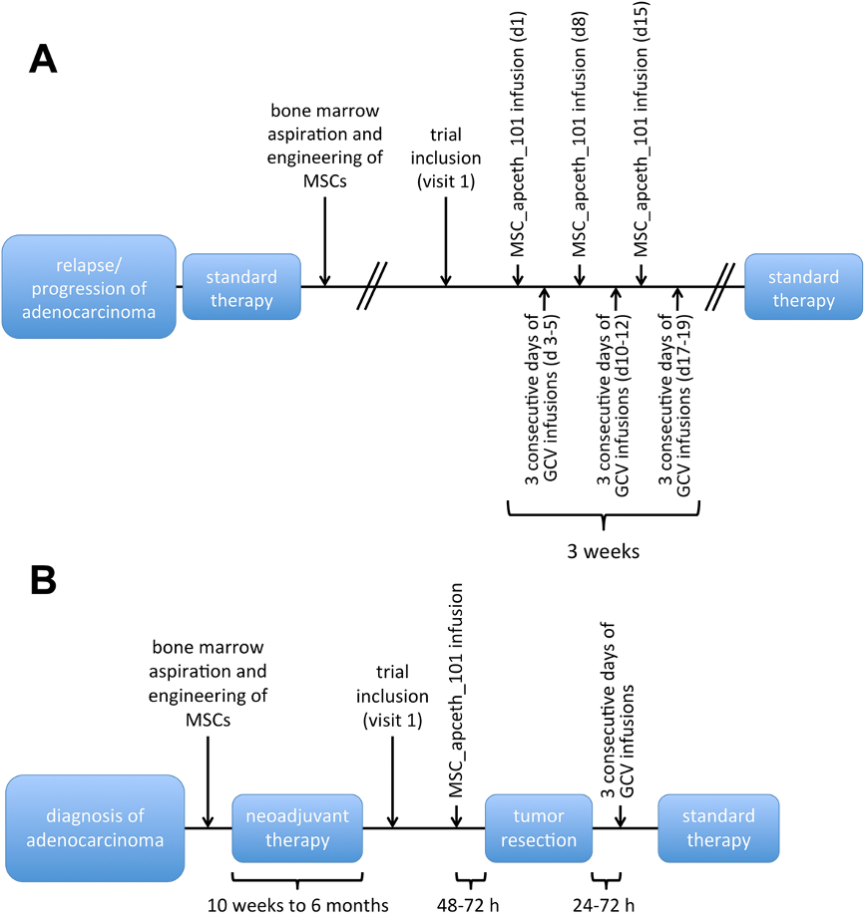
**Schematic view of the application sequences of the IMPs. (A)** Patients in group 1 of phase I or II will receive the IMPs in the sequence depicted here. **(B)** Group 2 of phase II, which consists of patient that receive neoadjuvant therapy for resectable tumors will receive the IMPs in the sequence portrayed here.

Figure 2 depicts the stages of the TREAT-ME1 study. In phase I, the safety and tolerability of the IMP will be investigated in six patients. Three of the six patients will receive the cells at a total dose of 1.5 × 10^6^ cells/kg (dose 1), applied intravenously in three equal doses of 0.5 × 10^6^ cells/kg each one week apart. The exact application scheme is depicted in Figure 3A. The other three patients will receive three doses of 1 × 10^6^ cells/kg adding up to a total dose of 3 × 10^6^ cells/kg (dose 2) after obtaining approval for dose escalation by the independent DSMB (Data and Safety Monitoring Board). Injection of the IMP will be followed by intravenous GCV injection dosed according to the manufacturer’s recommendation, which will be at 5 mg/kg b.i.d. when a creatinine clearance of greater than 69 ml/min is present and 2.5 mg/kg b.i.d. if the creatinine clearance is reduced to 50–69 ml/min. Time interval between GCV administrations will be 12 ± 3 hours. GCV will be given on three consecutive days starting between 48–72 hours after injection of MSC_apceth_101.

The rationale for the scheduling of MSC and GCV administration is based on the investigators preclinical studies, where these time intervals have proven optimal for ensuring that MSCs have enough time to enter the tumor stroma from the peripheral circulation and activate the therapeutic transgene [9, 10, 11, and unpublished data].

After all safety-relevant data of patients in phase I have been collected, an interim analysis will be performed. Safety-relevant data are serious and non-serious adverse events, including any clinically relevant changes of laboratory parameters or vital signs of patients. The safety data will be prepared for the data and safety monitoring board (DSMB) for review. The interim analysis along with the recommendation of the DSMB will be forwarded to the Paul-Ehrlich-Institute, the German national regulation authority for IMP trials, which is responsible for the evaluation of somatic and cell therapy products, and the leading ethics committee as a substantial amendment. Both institutions will evaluate whether the tolerability of MSC_apceth_101 in combination with GCV is sufficiently good to proceed to phase II.

In phase II, sixteen patients with adenocarcinoma from the digestive system will be enrolled in total. Patients will be treated with the cell dose determined safe in the interim analysis following phase I. They will be divided into two groups: Group 1 consists of patients with advanced disease as defined in phase I and will solely receive MSC_apceth_101 and GCV (Figure 2A). Group 2 comprises of patients with adenocarcinoma that qualify for surgery with prior neoadjuvant treatment. Here, bone marrow shall be drawn prior to the start of the neoadjuvant treatment. Then, during the course of the neoadjuvant treatment, MSC_apceth_101 will be produced. If the patient still qualifies for surgery after re-staging following neoadjuvant therapy, the respective patient will then be enrolled into the trial. MSC_apceth_101 will be administered as a single dose 48–72 hours prior to surgery according to the dose determined in phase I. On day 1–3 following surgery, patients will receive GCV infusions in order to abolish residual MSC_apceth_101 (Figure 3B).

### Clinical trial population

#### Inclusion criteria

Patients included in this trial, regardless of trial phase, must meet all of the inclusion criteria listed below:

1. Adequate organ function:
  - Hemoglobin > 9.0 g/dl
  - White blood cells > 3,000 cells/μl and Neutrophils > 500 cells/μl
  - Platelets > 100,000 cells/μl
  - Serum ALT/AST ≤ 3 x upper normal limit
  - Serum Total bilirubin ≤ 1.5 upper normal limit
  - Serum creatinine ≤ 2.0 mg/dl and creatinine clearance ≥ 50 ml/min
2. Anticipated minimal life expectancy of at least 6 months
3. Men and women of reproductive potential must agree to follow accepted contraception methods during treatment and for 3 months after completion of treatment.
4. Ability of patient to understand character and individual consequences of clinical trial
5. Age ≥ 18 years
6. Written informed consent must be available before any study specific procedure is performed

The following inclusion criteria must be fulfilled for patients in phase I or phase II, group 1:

1. Patients with advanced or recurrent or metastatic GI/HPB adenocarcinoma. Target indications are:
  - adenocarcinoma of the esophagus (cT3NxMx or cT4NxMx),
  - adenocarcinoma of the pancreas (cT3NxMx),
  - adenocarcinoma of the stomach (cT3NxMx or cT4NxMx),
  - intrahepatic cholangiocellular carcinoma (cT3NxMx or cT4NxMx)
  - metastasis from colorectal cancer (cTxNxM1)
2. Premature or scheduled termination of standard therapy (including possible preoperative therapy) due to intolerability/progress/inefficacy or no acceptance by the patient if relapses/metastases are measurable by means of imaging techniques. No relapse or metastases should exceed 5 cm in its maximal diameter.
3. In case of metastases from colorectal cancer:
  - Metastases are unresectable
  - The total volume of the liver metastases does not exceed 30 % of the total liver volume
  - In addition to liver metastases, lung metastases are tolerable, however not more than 10 in number with the largest not exceeding 10 mm in diameter
4. Patients have donated bone marrow for the production of MSC_apceth_101 based on a separate approved protocol. MSC_apceth_101 must have been released for the (autologous) use in the same patient.
5. The presence of progressive disease as clinically assessed by the investigator.

In addition to the before mentioned inclusion criteria, the following inclusion criteria must be fulfilled for patients in phase II, group 2:

Patients with advanced or recurrent or metastatic adenocarcinoma originating from the digestive system, where surgical resection may become an option but neoadjuvant therapy is necessary. Target indications are:

- Adenocarcinoma of the esophagus (cT3NxMx or cT4NxMx)
- Adenocarcinoma of the pancreas (cT3NxMx),
- Adenocarcinoma of the stomach (cT3NxMx or cT4NxMx),
- Intrahepatic cholangiocellular carcinoma (cT3NxMx or cT4NxMx)
- Colorectal carcinoma (cT2NxM1, cT3NxMx, cT4NxMx)

### Exclusion criteria

All patients enrolled in this trial independent of phase/group must not fulfill any of the following criteria:

1. Patients with severe heart disease (NYHA stage III/IV; unstable angina pectoris or myocardial infarction during the last 8 weeks before Visit 1 (baseline)
2. Clinically significant ischemic disease during the last 4 weeks before Visit 1
3. Severe lung disease (COPD GOLD grade III/IV, asthma bronchiale grade IV, lung fibrosis)
4. General condition poorer than ECOG2
5. Clinically proven peritoneal carcinomatosis (e.g. by the presence of ascites)
6. Clinically proven significant pleural/pericardial effusion
7. Serious uncontrolled acute infections less than 3 weeks before Visit 1
8. Patients with HBV/HCV infections
9. Patients with HIV infection
10. Immunodeficiencies or systemic autoimmune diseases (e.g. Crohn’s disease) or known malformations of the gastrointestinal tract
11. Active HSV infections by IgM positivity
12. Second carcinoma in addition to the underlying carcinoma unless the second carcinoma was curatively and successfully resected more than 5 years before Visit 1 (exception: basal cell carcinoma of the skin, precancerous lesions)
13. Use of any immunomodulators
14. Need for chemotherapy or radiotherapy or cytokine treatment (e.g. interferons, G-CSF or GM-CSF) in the time interval 2 weeks before infusion of MSC_apceth_101 or anticipation of chemo- or radiotherapy during treatment with MSC_apceth_101 and GCV until 2 days after the last administration of GCV
15. Known addiction to alcohol or drugs
16. Patients requiring corticoids in doses above the Cushing threshold
17. Known liver fibrosis or cirrhosis
18. Any concomitant severe disease which could compromise the objectives of this study in the judgment of the investigator
19. Female patient who is pregnant or breast feeding
20. Participation in another clinical trial or observation period, respectively, during the last 4 weeks prior to the first MSC_apceth_101 dose
21. Any surgery in the last four weeks before the administration of MSC_apceth_101
22. History of hypersensitivity to the investigational product or to GCV, Valganciclovir, Aciclovir or Valaciclovir
23. Any contraindication to Ganciclovir according to the product information or the need for drugs interacting with GCV

### Objectives and endpoints

Objectives and endpoints of the TREAT-ME1 study are summarized in Table 1.

**Table 1:** **Objectives and endpoints of the TREAT-ME1 study**

Objective	Endpoint
Primary	Safety and tolerability of MSC_apceth_101 (phase I and II)
Key secondary	• Total and individual size of relapse/metastases by CT or MRT according to RECIST criteria (phase I/II, group 1)
	• Detection of the therapeutic transgene expressed by homing MSC in resected tumor specimen by PCR technique shortly after its administration (phase II, group 2)
	• For comparison: Detection of the therapeutic transgene expressed by homing MSC in normal tissue adjacent to the tumor (phase II, group 2)
	• Optionally: PET activity in initially PET positive patients (phase I/II, group 1)
	•Tumor/serum markers (phase I/II, both patient groups)
Other secondary	•Time to progression up to 1 year after first MSC_apceth_101 administration (phase I/II)
	• Overall survival up to 1 year after first MSC_apceth_101 administration (phase I/II)

The Safety Set (SAF) of patients, which will be used for analysis of all safety-related data, will include all patients who received at least one dose of MSC_apceth_101. The Full Analysis Set (FAS) will consist of all patients who received at least one dose of MSC_apceth_101 and have at least one disease assessment. Safety-relevant data to determine the primary endpoint of this trial will include reporting of any adverse event, laboratory and other safety parameters determined in the trial visits.

### Investigation schedule, follow-up and assessments

Tables 2 and 3 illustrate the full investigational schedules for groups 1 and 2, respectively.

**Table 2:** **Investigational schedule for patients in group 1 of phase I and II**

Consultation visit	1	2, 6, 10	3, 7, 11	4, 8, 12	5, 9, 13	14	15	16	17	18
Days	(−10) to (−3)	1/8/15	3/10/17	4/11/18	5/12/19	29±3	56±7	168±14	252±14	336±14
**Parameter**	**Screening** ^**1**^	**MSC_apceth_101 infusion** ^**2**^	**GCV infusions** ^**3**^							
**Informed consent**	X									
**Inclusion/exclusion criteria**	X									
**Medical history/demography**	X									
**Physical examination**							X			
**Adverse events**		X	X	X	X	X	X			
**Concomitant medication**	X	X	X	X	X	X	X			
**Vital signs**	X	X^4^	X^5^	X^6^	X	X	X			
**Body weight**	X	X^7^				X	X			
**12-lead ECG**	X					X	X			
**Cardiac monitoring**		X^8^	X^9^	X^10^						
**Oxygen saturation**		X^8^	X^9^	X^10^		X				
**Tumor markers** ^**11**^	X						X			
**Tumor status**	X						X			
**MSC_apceth infusion**		X								
**GCV infusion**			X^12^	X	X					
**MRT or CT (PET optional)**	X^13^						X^14^			
**QoL assessment**	X					X	X			
**Serum pregnancy test**	X						X			
**Urine analysis**	X						X			
**Hematology**	X	X			X	X	X			
**Blood chemistry**	X	X	X	X	X	X	X			
**Creatinine clearance**	X									
**Inflammation markers**	X					X	X			
**Coagulation markers**	X	X			X	X	X			
**Infectious disease markers**	X						X			
**Lymphocyte subsets**	X					X	X			
**Biomarker samples**		X	X	X^10^						
**Retention samples**		X7				X	X			
**Immunogenicity samples**	X					X				
**ECOG**	X						X			
**Disease status** ^**15**^								X	X	X

Footnotes:^1^ not earlier than two weeks after end of chemo-/radiotherapy; ^2^ 24 h (±2 h) inpatient monitoring after end of MSC_apceth_101 infusions;^3^ 24 h (±3 h) inpatient monitoring after start of 1^st^ GCV infusion and including the 3^rd^ GCV infusion (discharge from hospital 2 h after 3^rd^ infusion); ^4^ Before and 15,30,60 min, 2 h, 4 h (±0.5 h), 6 h (±1 h), 8 h (±1 h), 12 h (±3 h), and 24 h (±2 h) after the end of MSC_apceth_101 infusion; ^5^ Before and 15,30,60 min, 2 h, 4 h (±0.5 h), 6 h (±1 h), 8 h (±1 h) after start of 1^st^ GCV infusion and before and 15, 30, 60 min and 2 h after 2^nd^ GCV infusion;^6^ Before and 15, 30, 60 min and 2 h after each GCV infusion; ^7^ Before MSC_apceth_101 infusion; ^8^ Before and 6 h (±1 h) and 24 h (±2 h) after start of infusion of MSC_apceth_101; ^9^ Before and 6 h (±1 h) after start of first infusion of GCV; ^10^ 1.5 h (±0.5 h) after 3^rd^ GCV infusion; ^11^ tumor marker: CEA, CA15-3, CA19-9, CA125, CA72-4, Cyfra21-1, AFP; only tumor marker applying for the tumor entity are determined;^12^ First GCV infusion not earlier than 48 h after preceding MSC_apceth_101 infusion; ^13^ Scans not older than 4 weeks can be used as baseline scans;^14^ MRT or CT or PET whatever was used at visit 1; repeat PET only if PET was done at visit 1; MRT or CT or PET can be carried out up to 28 days after day 56 if this is more convenient for the procedures in the hospital. ^15^ Survival status, any new relapses/metastasis, ECOG, treatment history: chemotherapy, radiotherapy, any other antitumor treatment.

**Table 3:** **Investigational schedule for patients in group 2 of phase II**

Consultation visit	1	2	3	4	5	6	7	8	9	10	11^1^	12	13	14
Days	(−10) to (−3)	1	3 or 4	5	6	7	8	11	16±2	22±2	29±3	168±14	252±14	336±14
Parameter	Screening^2^	MSC infusion^3^	Tumor resection	GCV infusions^4^										
**Informed consent**	X													
**Inclusion/exclusion criteria**	X													
**Medical history/demography**	X													
**Physical examination**	X										X			
**Adverse events**		X	X	X	X	X	X	X	X	X	X			
**Concomitant medication**	X	X	X	X	X	X	X	X	X	X	X			
**Vital signs**	X	X^5^		X^6^	X^7^	X^7^	X		X		X			
**Body weight**	X	X^8^		X^9^			X		X		X			
**12-lead ECG**	X						X				X			
**Cardiac monitoring**		X^10^		X^11^	X^12^									
**Oxygen saturation**		X^13^		X^11^	X^12^		X							
**Tumor marker** ^**14**^	X								X		X			
**Tumor status**	X										X			
**MSC_apceth infusion**		X												
**Tumor resection** ^**15**^			X											
**GCV infusion**				X	X	X								
**Serum pregnancy test**	X										X			
**Urine analysis**	X								X		X			
**Hematology**	X	X		X			X		X		X			
**Blood chemistry**	X	X		X			X		X		X			
**Creatinine clearance**	X													
**Inflammation markers**	X								X		X			
**Coagulation markers**	X	X		X^9^			X		X		X			
**Infectious disease markers**	X										X			
**Lymphocyte subsets**	X										X			
**Biomarker samples**	X										X			
**Retention samples**		X^8^					X		X		X			
**Immunogenicity samples**	X										X			
**ECOG**	X										X			
**Disease status**												X	X	X

Footnotes:^1^ in case subsequent chemo- or radiotherapy begins before day 29 all investigations of this day are to be carried out up to 5 days before therapy starts. Thereafter, no further AE recording is required; ^2^ not earlier than 2 weeks after end of chemo-/radiotherapy; ^3^ 24 h (±2 h) inpatient monitoring after end of MSC infusion; ^4^ 24 h (±3 h) inpatient monitoring after start of 1^st^ GCV infusion and including the 3^rd^ GCV infusion; ^5^ Before and 15, 30, 60 min, 2 h, 4 h (±0.5 h), 6 h (±1 h), 8 h (±1 h), 12 h (±3 h), and 24 h (±2 h) after the end of MSC_apceth_101 infusion; ^6^ Before and 15, 30, 60 min, 2 h, 4 h (±0.5 h), 6 h (±1 h), 8 h (±1 h), 12 h after start of 1^st^ GCV infusion and before and 15, 30, 60 min and 2 h after 2^nd^ GCV infusion; ^7^ Before and 15, 30, 60 min, 2 h after the start of each GCV infusion; ^8^ before MSC infusion; ^9^ before 1^st^ GCV infusion; ^10^ Before and 6 h (±1 h) and 24 h (±2 h) after end of MSC infusion; ^11^ Before and 6 h (±1 h) after start of 1^st^ GCV infusion; ^12^ 1.5 h (±0.5 h) after start of 3^rd^ GCV infusion; ^13^ Before and 6 h (±1 h) and 24 h (±2 h) after end of insufion of MSCs; ^14^ tumor marker: CEA, CA15-3, CA19-9, CA125, CA72-4, Cyfra21-1, AFP; only tumor marker applying for the tumor entity are determined; ^15^ Including biopsy of surrounding healthy tissue (will be used to detect MSCs) and should be at least 48 h after MSC infusion.

For the assessment of quality of life, the EORTC QLQ-PAN26 and QLQ-C30 (version 3.0) will be used. The EORTC QLQ-C30 is a questionnaire to measure general quality of life in cancer patients. The questionnaire consists of 9 multi-item scales: five functional scales (physical, role, cognitive, emotional and social), three symptom scales (fatigue, pain and nausea/vomiting) as well as global health and quality of life scales [19]. Specific symptoms including dyspnea, insomnia, anorexia, constipation, diarrhea and financial impact are measured as six single items. The EORTC QLQ-C30 has been used extensively in studies on cancer patients and has proven good internal consistency (alpha > 0.70) and good test-re-test reliability (0.80-0.90) [20].

In patients undergoing surgical resection (group 2), all surgical specimens will be analyzed for the presence of tissue-related and response predictive markers, i.e. the presence of MSC and the transgene, the activation of any transgenic promoter or the transcription of the therapeutic gene as well as the presence of other surrogate markers like tumor neoangiogenesis, the inflammatory tumor microenvironment (iTME) and related factors (e.g. macrophages, TNF-alpha, IFN-gamma etc.). The specific co-localization of the modified autologous cells to the tumor site can be demonstrated by immunohistochemistry or PCR.

### Adverse events (AE)

Registration of any adverse events (AE) following administration of the IMP is a key factor for evaluation of the safety of the IMP. AEs will be defined and evaluated regarding their severity according to the Common Terminology Criteria of Adverse Events (CTC-AE). An AE is defined as the appearance of (or worsening of any pre-existing) undesirable sign(s), symptom(s), or medical condition(s) that occur after a patient received the first dose of IMP and during the following time periods:

- For phase I and phase II, group 1: from Visit 2 until Visit 15. If any radio- or chemotherapy starts before day 56 the reporting of AEs will be stopped.
- For phase II, group 2: from Visit 2 until Visit 11 or until start of any radio- or chemotherapy.

It has to be taken into consideration that the safety data evaluation of patient group 2 will be influenced and superimposed by the concomitant surgery and medication and potential other related procedures. The investigator will be asked to evaluate the causal relationship of AE to surgery and/or associated concomitant procedures/medications or to the IMP diligently.

Serious adverse events (SAE) are defined as AEs meeting at least one of the following criteria:

- Is fatal or life-threatening
- Results in persistent or significant disability/incapacity
- Constitutes a congenital anomaly/birth defect
- Is medically significant, i.e. defined as an event that jeopardizes the patient or may require medical or surgical intervention to prevent one of the outcomes listed above
- Requires inpatient hospitalization or prolongation of existing hospitalization

To ensure patient safety, every SAE regardless of suspected causality, occurring after the patient received the first IMP administration and during the safety time periods as indicated above must be reported in writing within 24 hours of first knowledge of its occurrence. Any SAE arising outside the safety time periods should only be reported if the investigator suspects a causal relationship to the IMP or Ganciclovir.

### Safety follow-up

The safety follow-up is not limited to 56 days and 29 days from the IMP administration in group I and II, respectively. Apart from day 56, there are further safety follow-up visits to evaluate the long-term effects. At any time also unscheduled visits are possible.

The safety follow-up visits include visit 16 on day 168 (±14 days), visit 17 on day 252 (±14 days), and visit 18 on day 336 (±14 days). During these visits the disease status, ECOG status, and treatment history for chemotherapy, radiotherapy, surgery, other anti-tumor treatments will be evaluated (see Tables 1 and 2).

## Discussion

The significance of the surrounding stroma on tumor growth has become a focus of cancer research in recent years. In accordance with the results obtained by other groups, our preclinical studies showed incorporation of circulating MSCs into the tumor stroma [21]. As first envisioned by Studeny et al., MSCs can successfully be used as vehicles for delivery of anti-tumor agents [8]. We have utilized this capacity of MSCs and developed a combination of cell- and gene therapy by engineering bone marrow derived MSCs to express the suicide-gene *HSV-Tk*. Expression of this suicide gene was put under the control of a promoter we presume to be activated in MSCs specifically after homing into the tumor stroma. This step was implemented in order to lower systemic side effects.

The TREAT-ME1 trial was conceived based on the previously performed preclinical studies. In this trial, bone marrow from each enrolled patient is used to produce the investigational medicinal product (IMP), which consists of genetically engineered MSCs, termed MSC_apceth_101. This gene therapy medicinal product (GTMP) has undergone extensive preclinical studies and a risk evaluation according to the respective guidelines of the European Medicines Agency (EMA/EMEA) and the Committee for Medicinal Products for Human Use (CHMP) [22]. The gamma-retroviral, replication-incompetent and self-inactivating vector system we used to generate the MSC_apceth_101 resulted in a high degree of safety in these pre-clinical studies, especially with regards to the known potential risk factors that GTMPs inherit and that are defined by the respective EMA guideline [23]. The autologous cell batch produced for each individual patient undergoes extensive safety and quality testing before release, as described in the methods section of this manuscript. Federal authorities (Paul-Ehrlich-Institut) positively evaluated the safety profile and risk evaluation of the GTMP and a production license was issued. Furthermore, the concerned ethics committee approved this study protocol and patients are currently being recruited for the trial.

MSC_apceth_101 target he tumor stroma as treatment and – if well tolerated and high in efficacy – could be well combined with conventional therapies such as chemo- or radiotherapy while maintaining a low side effect profile due to the syngeneicity of the MSCs used and the tumor specific expression of the therapeutic gene construct. To our knowledge, the TREAT-ME1 trial is the first clinical trial worldwide using genetically altered MSCs in humans. Therefore, the primary endpoint of this trial is safety of the combined cell and gene therapy applied here. Secondary endpoints include efficacy of the cell therapy, overall survival and time to progression as well as proof of MSC_apceth_101 integration and transgene expression inside the tumor.

## Abbreviations

AE: Adverse events
ALT: Alanine aminotransferase
AST: Aspartate aminotransferase
BUN: Blood urea nitrogen
CD: Cluster of differentiation
CT: Computed tomography
CTC-AE: Common Terminology Criteria of Adverse Events
DSMB: Data and safety monitoring board
ECG: Electrocardiogram
ECOG: Eastern Cooperative Oncology Group
EMA/EMEA: European Medicines Agency
EORTC: European Organisation for Research and Treatment of Cancer
EU-CTR: EU Clinical Trials Register
Eudra CT: European Union Drug Regulating Authorities Clinical Trials Database
G-CSF: Granulocyte-Colony Stimulating Factor
GCP: Good clinical practice
GCV: Ganciclovir
GI: Gastrointestinal
GM-CSF: Granulocyte macrophage Colony-Stimulating Factor
GMP: Good manufacturing practice
GTMP: Gene therapy medicinal product
HPB: Hepatopancreatobiliary
HSV: Herpes simplex virus
IMP: Investigational medicinal product
MRI: Magnetic resonance imaging
MSC: Mesenchymal stromal cells
PEI: Paul-Ehrlich-Institute
PET: Positron emission tomography
RECIST: Response Evaluation Criteria In Solid Tumors
SAE: Serious adverse events
SIN: Replication-incompetent and self-inactivating vector
Tk: Thymidine kinase
